# Effect of melatonin on stress-induced hyperglycemia and insulin resistance in critically-ill patients: A randomized double-blind, placebo-controlled clinical trial

**DOI:** 10.22088/cjim.13.1.51

**Published:** 2022

**Authors:** Fahimeh Naderi-Behdani, Fatemeh Heydari, Shahram Ala, Siavash moradi, Saeid Abediankenari, Hossein Asgarirad, Elmira khodabakhsh

**Affiliations:** 1 Department of Clinical Pharmacy, Faculty of Pharmacy, Mazandaran University of Medical Sciences, Sari, Iran; 2 Department of Anesthesiology and Critical Care Medicine, Imam Khomeini Hospital, Mazandaran University of Medical Sciences, Sari, Iran; 3 Educational Development Center, Mazandaran University of Medical Sciences, Sari, Iran; 4 Department of Immunology, Faculty of Medicine, Mazandaran University of Medical Sciences, Sari, Iran; 5 Department of Pharmaceutics, Faculty of Pharmacy, Mazandaran University of Medical Sciences, Sari, Iran; 6 Department of General Surgery, Imam Khomeini Hospital, Mazandaran University of Medical Sciences, Sari, Iran

**Keywords:** Stress-induced hyperglycemia-insulin resistance-Melatonin- APACHE II- ICU

## Abstract

**Background:**

Hyperglycemia is a common finding which is associated with increased mortality and morbidity among critically ill patients. There is currently no evidence that melatonin could improve stress induced hyperglycemia (SIH). In this study, we evaluated the effect of melatonin on blood sugar and insulin resistance (IR) in critically-ill patients.

**Methods:**

104 critically-ill patients with SIH divided into two groups, receiving melatonin (6 mg BD for 3 days) or placebo. Changes of blood sugar, IR indices including homeostasis model assessment for insulin resistance and homeostasis model assessment adiponectin (HOMA-AD) ratios, Glasgow coma scale (GCS) were evaluated on the 4^th^ day of melatonin prescription. On the 7^Th day ^of study, changes of ventilator dependency and delirium were considered. Mortality and intensive care unit (ICU) stay were also compared between groups.

**Results:**

On day 4, patients in the melatonin group had significantly lower blood glucose and HMOA-IR level compared with the placebo group (P=0.04 and P=0.03, respectively) whereas HOMA-AD level did not differ significantly from placebo group (p>0.2). Also, we did not observe any significant difference in GCS level at this time between groups (p>0.2). On day 7, melatonin could not improve ventilator dependency and delirium significantly (p>0.2) and also could not reduce mortality and ICU stay in comparison with placebo (p>0.2, P=0.2, respectively).

**Conclusion:**

Melatonin supplementation showed positive effect on blood sugar and somehow insulin resistance whereas it could not improve ICU complications.

Stress-induced hyperglycemia (SIH) is defined as temporary insulin-resistance associated with increased production of glucose and lack of sufficient insulin secretion in non-diabetic patients. It can be developed by surgery or other medical reasons (1) and is linked to higher incidence of mortality and morbidity (1, 2). It generally occurs in the first 48 hours of hospitalization in the intensive care unit (ICU) (3). American Diabetes Association (ADA) has defined SIH as a random blood sugar above 140 mg/dl (7.8 mmol/L) (4). Although SIH will be resolved if the stress inducing factors or surgery effects are eradicated, many affected patients experience carbohydrate intolerance or diabetes in the follow-up visits (5, 6). However, insulin infusion has been the preferred therapeutic approach for the management of SIH for many years. Unfortunately, attempts to overcome SIH could induce hypoglycemia that is associated with high mortality rate (7-9). 

In intensive care units, insulin is administered to these patients, which has been beneficial in reducing the mortality rate, but for a short time (10). On the other hand, melatonin has been effective on glucose metabolism in humans and diabetic patients has shown lower melatonin level at night which confirms the correlation between melatonin and hyperglycemia (11). Melatonin has been found to have both positive effects on ßcells’ survival in pancreas and stimulatory effects on insulin secretion in rats (12). Furthermore, melatonin prevents accumulation of oxygen reactive species (ROS). Its effect on hyperglycemia could be explained not only by carbohydrate metabolism regulation but also by its antioxidant properties (13, 14). 

However, studies have demonstrated contradictory findings related to melatonin effect on carbohydrate metabolism, so we have not yet reached a definite opinion (15). Human and animal studies have acknowledged that short-term exogenous melatonin consumption is safe even at high doses (16). Moreover, it has also improved the mechanical ventilation time and Glasgow coma scale (GCS) level in ICU patients (17). On the other hand, the abnormal pattern of melatonin secretion can be considered as one of the leading causes of delirium in ICU that increases ventilator dependency and ICU stay. Delirium would be an independent risk factor for all-cause mortality. Yet there is not sufficient data on recommending exogenous melatonin in ICU (18). Hence, this study was accomplished to assess the role of melatonin in SIH and insulin resistance in critically-ill patients for the first time. The authors selected melatonin because of the blood sugar reducing effect of melatonin both in animal and laboratory studies, and the lack of sufficient data on its effect in humans as well as its beneficial effects in reducing mortality and morbidity rate.

## Methods

**Study design and ethical consideration: **This double-blind, placebo-controlled randomized clinical trial was conducted between October 2019 and July 2020 at General Intensive Care Unit of Imam Khomeini Hospital, a referral tertiary teaching hospital affiliated to Mazandaran University of Medical Sciences, Sari, Iran. The study was also approved by Institutional Review Board and the Ethics Committee of Mazandaran University of Medical Sciences (code number: IR.mazums.rec.1398.1260). Then, it was registered in the Iranian Registry of Clinical Trials Center with a code number of IRCT20100107003014N23.Written informed consent forms were obtained from either the patients or their care providers, if not feasible.

**Setting and patients: **Adult surgical, medical, and traumatic patients (age≥18 years old) who were admitted to ICU with two blood sugar more than 140 within the first 24 hours of admission were recruited. One of the blood sugar was measured exactly on admission period as random blood sugar, another one belonged to fasting blood sugar measurement of patient just before intervention. Patients with diabetes mellitus, hypersensitivity to different melatonin dosage forms, HbA1c >6.5, NPO drug state, and those receiving glucocorticoid therapy and dextrose serum at night, as well as pregnant women and patients who died or had early ICU discharge before completing the study, were excluded from the study. 

**Sample size and randomization: **Based on Patricia Rubio-Sastre et al.’s study that found out melatonin could change insulin serum 18 µU/mL with standard deviation (SD) of 33 µU/mL in comparison with placebo, power of 80%, and confidence interval of 95%, 47 patients in each group were determined. Considering a 10% attrition rate, 52 patients in each group were recruited.

Using the random block generator software, eligible patients were randomly assigned to two groups receiving either melatonin or placebo for three days. They were divided into 13 blocks of 8 individuals. All products in the study were completely identical in terms of appearance and packaging. The patients, the evaluator, and the prescriber were blind to the intervention allocation. Intervention group received melatonin tablet 3 mg (made by RAZAK Company) twice daily (12 AM and 12 PM) each time 2 tablets for 3 days. On the other hand, the control group received placebo tablets in the same way the intervention group received melatonin. Melatonin and placebo that were absolutely similar were placed in similar plastic cans and were coded as A A B B A A B B.

**Study intervention and outcome measurement: **A 5-ml peripheral venous blood sample was taken from each eligible patient before starting the intervention to measure serum glucose, insulin, adiponectin, and HbA1c. Three days after the intervention (the 4^th^ day); serum glucose, insulin, and adiponectin were measured again. All blood samples were collected at fasting state. The serum sample tubes were centrifuged for 10 minutes at 1000 rpm by BH-1200 model made in Iran. Glucose concentration was determined through isolated serum within 1 hour after blood sampling using a blood autoanalyzer. Serum aliquots were stored in the refrigerator at -80 until the end of the trial. Adiponectin and insulin serum were measured by enzyme-linked immunosorbent assay method(BIOTEK ELISA, elx800, USA) with commercial reagents (AD ELISA kit: Enzyme Immunoassay for quantitative Determination, Mediagnost, Germany; insulin ELISA kit: Direct Immunoenzymatic Determination, diametra, Italy) (1). Patients requiring parenteral nutrition were excluded from the study and fluids containing glucose were withheld at night due to fasting state. Different feeding types of the patients were listed by the evaluator to assess the effect of feeding at the end. In both groups, patients experiencing blood glucose level of above 200 mg/dl received regular insulin based on ICU protocol (BS-180/30). Dextrose infusion rate did not exceed 4mg/kg of body weight per minute to prevent infusion-related hyperglycemia. Insulin resistant indices including HOMA-IR (homeostasis model assessment of insulin resistance) and HOMA-AD (homeostasis model assessment-adiponectin) were calculated using the following formulas: HOMA-IR= (fasting insulin (mu/L) *fasting glucose (mmol/L))/ 22.5; HOMA-AD= (fasting insulin (mu/L) *fasting glucose (mmol/L))/ (22.5*fasting AD µg/ml) (19-21).

**Figure:**
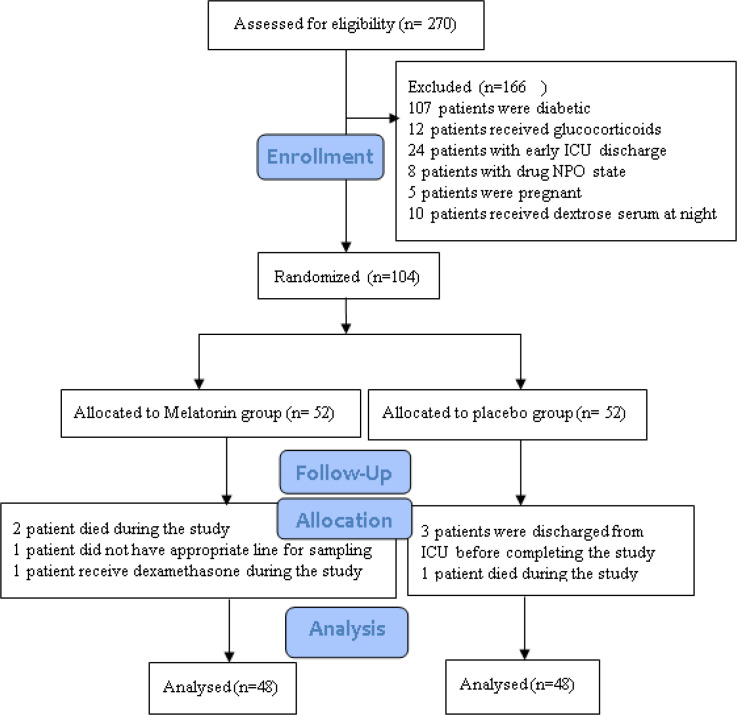
Consort diagram

Demographic characteristics consisting of age, sex, weight, height, reason for ICU admission, type of nutrition as well as the baseline laboratory data consisting of HbA1c, Hemoglobin (Hgb),white blood cell (WBC), platelets (PLT), alanine aminotransferase (ALT), aspartate aminotransferase (AST), alkaline phosphatase (ALP), bilirubin total, bilirubin direct, international normalized ratio (INR), urea, serum creatinine, GCS, and Acute Physiology and Chronic Health Evaluation II (APACHE II)were all recorded before the intervention. Consumed drugs of melatonin and placebo groups were compared between the groups. GCS was also measured after the intervention on the 4^th^ day of the study. Delirium and ventilator dependency were assessed before the intervention and on the7^th^ day of the study for those patients who remained in the ICU. Richmond Agitation Sedation Scale (RASS) was employed for delirium evaluation. Days of ICU stay and survival status up to hospital discharge were recorded for all analyzed patients. Any suspicious side effects induced by melatonin were also reported.

**Statistical analysis: **Statistical analysis was performed using SPSS Version 22. The Kolmogorov-Smirnov test was used to assess the status of distribution of quantitative variables. Quantitative variables including median (interquartile range) due to abnormal distribution of the data and qualitative variables including frequency and percentage were presented. To compare quantitative variables, independent sample Mann-Whitney U test was used and for assessing qualitative variables, we run Pearson’s chi-square or Fisher’s exact test. Wilcoxon test was also performed for within group analysis. A p<0.05 was considered statistically significant.

## Results

 Out of 270 patients who were screened for the inclusion/exclusion criteria, only 104 patients met the required criteria of the study and were randomized to receive either melatonin or placebo. 8 patients failed to accomplish the trial due to death (n=3), early ICU discharge (n=3), receiving dexamethasone during study (n=1), lack of appropriate peripheral line for blood sampling (n=1). Finally, 96 patients completed the study (Figure 1). The median age (IQR) of patients was 56 (44.00) years. 64.6% of patients (n=62) were males and 35.4 % (n=34) of them were females. 36.5 % (n=35) of patients with multiple trauma, 34.4 % (n=33) medical, 18.8% (n=18) closed traumatic brain injury (TBI), and 10.4 % (n=10) had cerebrovascular accident (CVA). Demographic, laboratory and clinical data of two groups was recorded at the time of ICU admission which is summarized in tables 1 and 2. There was no significant difference between two groups in admission period (p>0.05). Moreover, concomitant drugs were comparable between melatonin and placebo groups (p>0.2) (table 3). We did not observe any significant difference between the different types of feeding between the two groups (P=0.11) (table 1). 

**Table 1:** Baseline Demographic and Clinical Assessment of the patients

**characteristic**	**Melatonin Group** **(n=48)**	**Placebo Group** **(n=48)**	**P Value**
Age (years), median (IQR)	58 (33.75)	54 (33)	> 0.2
Sex, n (%)			
Male Female	33 (68.8) 15 (31.3)	29 (60.4) 19 (39.6)	> 0.2
BMI, median (IQR)	25.20 (4.62)	26.12 (5.20)	> 0.2
Cause of admission, n (%)			
MT Closed TBI Medical CVA	17 (35.4) 7 (14.6) 17 (35.4) 7 (14.6)	18 (37.5) 11 (22.9) 16 (33.3) 3 (6.3)	> 0.2
APACHE II score, median (IQR)	13(7.75)	11(9.75)	> 0.2
GCS, median (IQR)	10 (8.5)	11 (8)	0.16
Nutrition, n (%)			
Entera Meal gavage Hospital soup gavage Homemade gavage Po Npo	12(25) 5 (10.4) 2 (4.2) 21 (43.8) 8 (16.7)	5 (10.4) 2 (4.2) 4 (8.3) 21 (43.8) 16 (33.3)	0.11

IQR=interquartile range, BMI= body mass index, MT= multiple trauma, TBI= traumatic brain injury, CVA= cerebrovascular accident, APACHE II= acute physiology and chronic health evaluation, GCS= Glasgow coma scale, PO= by mouth, NPO= nothing by mouth

**Table 2:** Baseline Laboratory Parameters

**Variable, Median (IQR)**	**Melatonin Group** **(n=48)**	**Placebo Group** **(n=48)**	**P Value**
HbA1c, g/dL	5.5 (0.7)	5.3 (0.4)	> 0.2
Hgb, g/dL	11.15 (3.32)	10.7 (2.75)	> 0.2
WBC * 1000/µL	13.95 (8.25)	12.75 (7.37)	> 0.2
Platelet *1000/µL	202.5 (101.75)	174 (90.75)	> 0.2
Alanine aminotransferase unit/L	26.50 (39.5)	23 (25.5)	> 0.2
Aspartate aminotransferase unit/L	40.50 (59.75)	40.50 (42.5)	0.09
Alkaline phosphatase unit/L	210 (92.25)	192.50 (86.25)	> 0.2
BUN mg/dL	15 (9.87)	15 (10.75)	> 0.2
Serum creatinine concentration mg/dL	1 (0.4)	1 (0.37)	> 0.2
INR	1.10 (0.3)	1.10 (0.27)	> 0.2
Bilirubin total, mg/dL	1 (0.6)	1.2 (0.65)	0.10
Bilirubin direct, mg/dL	0.2 (0.2)	0.3 (0.2)	0.11

HbA1c= hemoglobin A1C= glycosylated hemoglobin, Hgb= hemoglobin, WBC= white blood cell, BUN= blood urea nitrogen, INR=international normalized ratio

**Table 3:** concomitant medications

**medication**	Melatonin group* **(n=48)**	Placebo group* **(n=48)**
H2 blocker	4	2
Anticonvulsant	27	25
Cephalosporines	30	33
Catecholamines	7	5
DVT prophylaxis	14	16
Proton pomp inhibitor	42	46
Diuretics	21	19
Vancomycin	4	4
Carbapenems	4	3
Insulin	5	4

H2 blocker=histamine Type-2 receptor antagonists, DVT prophylaxis= deep vein thrombosis prophylaxis

*p>0.2

There were no statistically significant differences in the serum glucose, insulin and adiponectin level and also insulin resistance parameters including HOMA-IR and HOMA-AD before intervention between the groups. We also did not detect any significant differences in secondary outcomes (GCS, APACHE II, delirium, ventilator dependency) at baseline between groups (P=0.15, p>0.2 respectively). Reduction in fasting serum glucose levels was detected on the 4^th^ day in comparison on the 1^st ^day of study in both groups (p<0.001). There was significant reduction in fasting serum insulin after intervention in both melatonin and control groups (p<0.001 and P=0.04, respectively). Fasting serum adiponectin levels increased significantly in the melatonin group (P=0.007) but not in the placebo group (P=0.20). Significant reduction in HOMA-IR ratio was observed in melatonin and placebo groups on day 4 of the study (p<0.001, P=0.002, respectively). Finally, decline in HOMA-AD ratio was significant in both groups (p<0.001, P=0.01 in melatonin and placebo groups, respectively). GCS reached statistical significant level in both the melatonin and control groups (p<0.001, P=0.003 respectively). Delirium amelioration in the melatonin group did not reach significant level (P=0.05) whereas, delirium deteriorated in the placebo group in a non-significant pattern (p>0.2). At day 4, patients in the melatonin group had significantly lower fasting blood glucose and HOMA-IR level compared with the placebo group (P=0.003 and P=0.04, respectively) whereas serum adiponectin, insulin and HOMA-AD level did not differ significantly (p>0.2, P=0.16, P=0.15, respectively). GCS also did not achieve significant difference (p>0.2) (table 4). At day 7, neither delirium, nor ventilator dependency were different between groups in a significant pattern (p>0.2) (tables 5 and 6). Comparison of ICU admission days did not present any significant difference between melatonin and placebo groups (P=0.2) and also the frequency of mortality was the same between groups (n=9, p> 0.2) (tables 5 and 6). The only side effect which could be attributed to melatonin was pruritus. The event was only seen in one patient in the melatonin group.

**Table 4:** Changes in GCS, APACHE II score and the glycemic indices during the study

**Variable**		**Melatonin group** **(n=48)**	**Placebo group (n=48)**	P-value*
		**MEDIAN(IQR)**	**MEDIAN(IQR)**	
Blood glucose, mg/dL	1^ th^ day	165 (29.5)	154 (42.5)	0.08
	4^th^ day	109 (26.25)	115.5 (37.5)	0.003
	P-value**	< 0.001	< 0.001	
Serum insulin, µU/mL	1^ th^ day	10.57 (17.65)	10.46 (15.46)	> 0.2
	4^th^ day	2.73 (8.76)	6.49 (9.47)	0.16
	P-value**	< 0.001	0.04	
Serum adiponectin, µg/mL	1^ th^ day	10.29 (10.47)	10.03 (9.87)	> 0.2
	4^th^ day	11.84 (11.3)	13.37 (12.05)	> 0.2
	P-value**	0.007	0.20	
HOMA-IR	1^ th^ day	4.54 (6.44)	4.40 (7.04)	>0.2
	4^th^ day	0.71 (2.31)	1.85 (3.1)	0.04
	P-value**	< 0.001	0.002	
HOMA-AD	1^ th^ day	0.51 (1.32)	0.31 (0.15)	0.17
	4^th^ day	0.08 (0.23)	0.13 (0.31)	0.15
	P-value**	< 0.001	0.01	
GCS	1^ th^ day	10 (8.5)	11 (8)	0.15
	4^th^ day	13 (9)	15 (7)	> 0.2
	P-value**	< 0.001	0.003	

*Mann Whitney U Test, **Wilcoxon Test

IQR= interquartile range, HOMA-IR= homoeostasis model assessment-insulin resistance, HOMA-AD= homeostasis model assessment-adiponectin ratio, GCS= Glasgow coma scale

**Table 5:** Assessment of secondary outcomes during the study

**Variable**		**Melatonin group** ** (n=48)**	**Placebo** **group (n=48)**	P-value*
		Median (IQR)	Median (IQR)	
Delirium	1^ th^ day	-2 (5)	0 (4.75)	> 0.2
	7^ th^ day	0 (4)	-3 (4.5)	> 0.2
	P-value**	0.05	> 0.2	
ICU stay		6 (9.75)	5 (6)	0.2

*Mann Whitney U Test, **WILCOXON TEST IQR= interquartile range, ICU= intensive care unit 7 ^Th ^day: Melatonin group (n= 23), placebo group (n=17)

**Table 6:** Comparison of ventilator dependency and mortality between groups

**variable**	**Melatonin group (n=48)**	**Placebo group (n=48)**	P value*
Ventilator dependency 1^ th^ day, n (%)	30 (62.5)	23 (47.9)	> 0.2
Ventilator dependency 7^ th^ day, n (%)	11 (47.8)	10 (55.6)	> 0.2
mortality	9 (18.8)	9 (18.8)	> 0.2

* Chi-Square Tests

7^Th ^day: Melatonin group (n= 23), placebo group (n=17)

## Discussion

SIH is a secondary response to stress in critically-ill patients as a consequence of surgery, TBI, and other medical illnesses. This condition is associated with remarkable morbidity and mortality rate. Moreover, SIH resulted a threefold higher death among traumatic non-diabetic patients (22, 23).

SIH induces many complications like type 2 diabetes (approximately double than those without this disease) in the long run, critical illness polyneuropathy (CIP), increased risk of mechanical ventilation, long stay in ICU, and infection (3, 24, 25). When the patients encounter an emergency such as sepsis, trauma, burn, or other stressful medical conditions, the normal homeostatic processes of body are disturbed. A combination of counter regulatory hormones (e.g., glucagon and growth hormone) and proinflammatory cytokines such as tumor necrosis factor alpha (TNF-α), interleukin-1 (IL-1), and interleukin-6 (IL6) lead to insulin resistance and hyperglycemia (26, 27). Therefore, attempts to overcome SIH consist of using agents such as insulin, metformin, vitamin D, and magnesium which are beneficial in reducing insulin resistance and inflammatory pathways (1, 28-30). Insulin can be an ideal therapeutic option for critically-ill patients especially those who are hemodynamically unstable (25). Tight glycemic control with insulin could worsen the outcome in patients with hypoglycemic episodes (30).

The indoleamine hormone; melatonin is produced by pineal gland and has essential role in circadian rhythm regulation. Melatonin has MT1 and MT2 receptors on islet beta cells of pancreas. It also plays an antioxidative role by free radical scavenging; hence, we expect beneficial effects of this supplement on reducing insulin resistance and glucose intolerance (13). Evaluating the influence of melatonin in experimental animal models (in vivo) presented regulation of glucose hemostasis; furthermore, it could attenuate insulin resistance in diabetic fatty rats via conquering mitochondrial dysfunction. Moreover, investigators have shown various effects of melatonin on glucose metabolism in humans. (31, 32). It has been revealed that diabetic patients manifested lower level of serum melatonin which indicates the existence of a link between melatonin and hyperglycemia (11).

The combination of melatonin and zinc acetate with or without metformin ameliorated fasting blood glucose and postprandial glycemic control,though it could not affect C-peptide in diabetic patients (33). Furthermore,it has been demonstrated that prolonged-release melatonin had long-term beneficial effects on glycemic control in this population(34). On the other hand, there are studies, in contrast with our assumption, which have resulted in the decrease of insulin sensitivity and glucose tolerance with acute administering of melatonin. Therefore, there is not an agreement about melatonin effects on glucose metabolism in humans (15, 35). Besides, faster rise in GCS and lower duration of mechanical ventilation in the melatonin recipients have been observed in ICU (17). Based on these findings, this study was designed to evaluate the efficacy of melatonin supplementation on FBS and insulin resistance parameters as primary outcomes and also; GCS, delirium, ventilator dependency, ICU stay, and mortality rate as secondary outcomes in critically ill traumatic and medical patients with SIH. Short-term use of exogenous melatonin is safe even in high doses, although some minor side effects such as headache, dizziness, nausea, and sedation have rarely occurred (16). In our study, 12 mg of melatonin oral daily (6 mg BD) was chosen because previous studies had also tried almost similar doses, i.e. 10 mg daily (35, 36). The only observed side effect of melatonin (pruritus) in our intervention resolved by administering antihistamine.

Due to short duration of SIH, included patients were followed-up for 3 days,as it would be eliminated if stress was removed (1, 28). HOMA-IR and HOMA-AD ratios were measured in this study as insulin resistance parameters. Insulin resistance is associated with hemodynamic changes and higher risk of cardiac metabolic dysfunctions. HOMA-IR is a strong surrogate marker for predicting IR. Euglycemic hyperinsulinemic clamp technique is a gold standard method for evaluating IR. However, this is not routinely used because of being costly and complicated. On the other hand, some studies claim that HOMA-IR is insufficient to detect insulin resistance particularly in diabetic patients (37, 38). Adiponectin is a kind of protein, which is made almost by adipose tissue and then secreted into the serum. This protein is decreased in insulin resistance condition(39). HOMA-AD is a modified version of HOMA-IR including adiponectin in the denominator of fraction. This parameter has an inverse correlation with insulin sensitivity. 

Previous studies have not achieved a consistent result regarding the superiority of this ratio in comparison to HOMA-IR (40-42). Melatonin consumption in the long run can improve HOMA-IR marker in polycystic ovary syndrome (PCO) patients and HbA1c in diabetic patients (34, 36). But there are not any data regarding its benefits to insulin resistance in short time administration. There are several factors which can affect blood glucose and insulin resistance parameters such as type of feeding, illness, severity of the disease, and hemodynamic condition (29). 

There were no significant differences between the two groups (melatonin and placebo) in the baseline characteristics, reason for admission, nutrition, Glasgow coma scale, and APACHE II score in our study. All blood sampling was done in the fasting state; therefore, we could unify our two groups and minimize the bias in the trial. In addition, recent studies have supported its favorable effects on neuronal survival and enhanced neurogenesis. Melatonin as a neuroprotective agent has the ability to improve the outcomes of patients with intracranial hemorrhage (ICH) via decreasing harmful consequences of hematoma. (43, 44). There is a higher rate of oxidative damage in non-surviving patients than surviving patients of ICH and higher level of endogen melatonin, which has been produced to overcome oxidative stress, but this higher serum melatonin level is insufficient to overcome high oxidative products (45). Based on these findings and other practical effects of melatonin observed in ICU, (17, 18, 46, 47)GCS was measured on the fourth day of the study. On the 7^th^ day, we compared delirium and delirium management (47, 48) and its facilitating effect on weaning process. We also evaluated mortality rate and length of ICU stay based on the advantages of melatonin supplementation on these items (49).

In this study, patients with SIH in the melatonin group showed significantly lower blood glucose and lower HOMA-IR level on the 4^th^day of the study in comparison with those in the placebo group. However, some conflicting results regarding the effects of melatonin on glucose tolerance and insulin resistance have been reported in the previous studies which may be attributed to low dose of melatonin, or small sample size. Moreover, the participants in the previous studies did not suffer from any acute illnesses involving stress (15, 34, 35). The previous studies conducted on PCO women and obese patients with acanthosis nigricans were in line with our trial in the reduction of HOMA-IR level by melatonin administration; however, both trials lasted 12 weeks (36, 50). In the present study, adiponectin level and HOMA-AD did not differ significantly between groups after melatonin administration, whereas anotherrstudy revealed that after melatonin supplementation, the adiponectin level significantly increased (51). GCS level was not significantly different between melatonin and placebo groups on the 4^th^day of the study that could be explained by the short duration follow-up of the patients and lack of intervention assignment to TBI patients exclusively (17). On the 7^th^day, delirium and ventilator dependency status did not improve significantly in the intervention group despite existing data that has confirmed its role in delirium management and decrease in ventilation time. There were also no statistically significant differences between the two groups in the mortality rate and ICU stay which may be attributed to the small sample size analyzed at the second time point (18, 47-49). This was the first study evaluating melatonin effects on SIH in critically ill patients. Potential limitations of our study need to be considered for future trials. First of all, our study assessed the patients for a short time. We also did not include a specific group of critically-ill patients such as TBI which could explain the lack of efficacy of melatonin on GCS. ICU complications did not change significantly due to small sample size in the follow-up. Multicenter, well-designed, randomized controlled trials by larger sample size are required to clarify the role of melatonin in critically-ill patients with longer follow-up.

In the present study, 12 Mg melatonin, oral daily for 3 days improved FBS and somehow insulin resistance in critically-ill patients with SIH, though it could not significantly improve ICU complications.

## Funding:

This study was the result of a residency thesis of Dr Fahimeh Naderi-Behdani supported by a grant from the Vice-Chancellery for Research and Technology of Mazandaran University of Medical Sciences.

## Conflict of Interests:

There is no conflict of interest
